# Variables Associated with the Use of Coercive Measures on Psychiatric Patients in Spanish Penitentiary Centers

**DOI:** 10.1155/2014/928740

**Published:** 2014-01-20

**Authors:** E. Girela, A. López, L. Ortega, J. De-Juan, F. Ruiz, J. I. Bosch, L. F. Barrios, J. D. Luna, F. Torres-González

**Affiliations:** ^1^Department of Legal and Forensic Medicine, Faculty of Medicine, University of Córdoba, Avenida Menéndez Pidal s/n, 14071 Córdoba, Spain; ^2^Medical Services, Secure Psychiatric Hospital of Alicante, Spain; ^3^Medical Services, Prison of Málaga, Spain; ^4^Medical Services, Prison of Córdoba, Spain; ^5^Medical Services, Prison of Granada, Spain; ^6^Medical Services, Secure Psychiatric Hospital of Sevilla, Spain; ^7^Department of Biostatistics, University of Granada, Spain; ^8^Center of Biomedical Research Network of Mental Health (CIBERSAM), University of Granada, Spain

## Abstract

We have studied the use of coercive medical measures (forced medication, isolation, and mechanical restraint) in mentally ill inmates within two secure psychiatric hospitals (SPH) and three regular prisons (RP) in Spain. Variables related to adopted coercive measures were analyzed, such as type of measure, causes of indication, opinion of patient inmate, opinion of medical staff, and more frequent morbidity. A total of 209 patients (108 from SPH and 101 from RP) were studied. Isolation (41.35%) was the most frequent coercive measure, followed by mechanical restraint (33.17%) and forced medication (25.48%). The type of center has some influence; specifically in RP there is less risk of isolation and restraint than in SPH. Not having had any previous imprisonment reduces isolation and restraint risk while increases the risk of forced medication, as well as previous admissions to psychiatric inpatient units does. Finally, the fact of having lived with a partner before imprisonment reduces the risk of forced medication and communication with the family decreases the risk of isolation. Patients subjected to a coercive measure exhibited a pronounced psychopathology and most of them had been subjected to such measures on previous occasions. The mere fact of external assessment of compliance with human rights slows down the incidence of coercive measures.

## 1. Introduction

The use of coercive measures is not uncommon in psychiatry, because of the frequent lack of insight on severe mental illnesses. In this context, the treatment of patients with mental disorders today runs up against the issue of patient autonomy [1] within an ethical framework [2, 3] in which paternalistic intervention has to be justified [4]. The boundaries demarcating a patient's ability to take responsibility for treatment are necessarily vague in the case of mental illness, and at times confused [5]. Patient consent is disregarded in many circumstances, either because it cannot be obtained in a valid manner or because measures may need to be adopted against the patient's will. Although, patients may be unaware of their illness, yet still they are able to take decisions regarding their treatment. In this respect, it should be borne in mind that the patient is not incapacitated “in general,” but only in terms of specific tasks and decisions. In nonpsychotic pathologies, such as eating disorders, drug dependency, or personality disorders, the problem becomes even more complex [6]. Moreover, coercion has a negative effect on the relationship between the patient and his or her carer [7].

The major implications of coercive measures have been a neglected issue in clinical psychiatric practice for some decades, and their use remains more common than being desirable [8]. The imperative need for research and the establishment of rules has only recently been recognised by member states of the European Union, through the EUNOMIA study [9]. As is evident from the results of this study, approximately one-third of involuntarily hospitalised patients are subjected to coercive measures such as restraint by holding and/or mechanical devices, isolation, or forced medication within the first four weeks of admission, although being with large variations between countries [10].

The issue of forced medication of prison inmates with psychiatric disorders is even more sensitive and even less investigated. It is clear, from the EUPRIS study, which compared 24 European countries [11], that patient consent is even more difficult to obtain in this context given that inmates are already deprived of their freedom, a fact which strongly influences compliance. Moreover, in some countries such patients are somewhat less protected than the general population in terms of the laws regulating compulsory treatments, especially when treatments are administered within the penal context. A set of essential mental health indicators in prison, highlighted in the EUPRIS final report, included an appropriate infrastructure for inpatients and outpatients, the importance of mental health training for medical staff working in prisons, and increased research into the prevalence of mental disorders in prisons and the mental health of inmates.

The present study (known by the acronym COCEHOSPE) sets out to examine the use of coercive measures for therapeutic purposes in prisons, following the same lines as earlier studies (EUNOMIA and EUPRIS). We have studied variables related to adopted coercive medical measures (forced medication, isolation, and mechanical restraint) in mentally ill inmates within two secure psychiatric hospitals (SPH) and three regular prisons (RP) in Spain.

## 2. Materials and Methods

Three prisons took part in this study: Málaga (Alhaurín de la Torre), Granada (Albolote) and Córdoba, each of which houses roughly 1500–1700 inmates. Two secure psychiatric hospitals (Sevilla and Alicante), with a capacity of 180 and 390 beds, respectively, also took part. Mentally ill persons are placed into secure psychiatric hospital whenever he/she is under criminal process, and the judge estimates that patient has lost the ability to act freely and/or patient does not understand the crime and its consequences. So it is strictly a criminal court decision, after taking into account medical expertise.

The study was undertaken with the authorization of the General Directorate for Penal Institutions and was carried out between January 2008 and February 2011.

The subjects of the study were patients who, in the course of a psychiatric disorder, had been subjected to one of the following coercive measures:

1. forced medication: prescribed and/or administered against the patient's will;
2. isolation: confinement in a room, restricting the freedom to leave it;
3. mechanical restraint: fixation of one or more limbs using mechanical devices.

The patients agreed to be included in the study, using an informed consent form, no more than two weeks after the episode involving coercive action.

A total of 209 patients (181 men and 28 women), with a mean age of 34.5, were studied. Of these, 108 patients were drawn from secure psychiatric hospitals and 101 from regular prisons.

The study was purely observational and sought to bring together a range of variables in a questionnaire similar to that used in the EUNOMIA study [9], although omitting all matters relating to involuntary admission and adapting it to the prison context. Briefly, the following data were collected:

- the subject: gender, age, marital status, data concerning the subject's legal and penal situation, sociodemographic data, and data relating to the subject's biological, psychological, and social wellbeing such as main diagnosis, history, and mental status: global assessment of functioning (GAF) [12] and brief psychiatric rating scale (BPRS) [13];
- the coercive measure: time, duration, frequency of supervision, the person who ordered or authorised the measure, and reporting of the measure in the case history, reporting of the measure to the judicial authorities;
- variables based on the subject's subjective opinion: personal view of the degree of coercion, overall perception of the treatment and care being received (CAT scale) [14];
- variables based on information supplied by staff: the reason for implementing the measure, the benefit sought, the level of patient aggression(modified overt aggression scale) [15], perception of the pressure exerted on the patient, and other relevant clinical data (degree of compliance with treatment, suspicions regarding drug consumption, etc.).

Data collection at each institution was always undertaken by the same observer (the researcher at the institution); this included the completion of scales. The observers previously received preparatory training by an expert in BPRS.

The statistical analysis was carried out using STATA 11.1 software. Descriptive variables (frequency distributions and basic summary measures) were analysed; a study was made of the agreement between the coercive measures as reported by patients and by medical staff, using the Kappa statistical test; finally, the factors associated with the use of each of the coercive measures were examined by means of univariate (chi-squared or Fisher's exact test for qualitative variables and Kruskal-Wallis test for quantitative variables) and multivariate techniques (logistical regression model).

## 3. Results

The patients' main characteristics are summarised in Table 1.

The main measure used in the episodes reported was isolation (41.35%), followed by mechanical restraint (33.17%) and forced medication (25.48%). In most cases (87%) the main coercive measure was accompanied or preceded by other measures: change of unit or cell in 120 cases (57.42%), solitary confinement in 64 (30.62%), immobilisation in 28 (13.4%), and forced medication in 66 cases (31.58%). In 15.31% of the cases the whole set of coercive measures was applied.

Comparison of the different coercive measures used revealed no statistically significant differences with respect to most variables, whether personal (age, gender, etc.), sociodemographic (nationality, etc.), mental health (the specific diagnosis was also nonsignificant), or penitentiary, although certain interesting differences emerged from multivariate analysis using the logistical regression model (see Table 2) with regard to the following factors: type of institution, existence of previous custodial sentences, previous episodes of psychiatric hospitalisation (not secure), existence of a live-in partner, and prisoner communications with family members.

In relation to patient pathological profiles depending of the type of institutions, we noticed that there was a statistical difference (*P* = 0.004) both in psychotic disorders (more frequent in SPH) and in substance-related disorders (less frequent in SPH).

We also found different profiles of results for the psychometric scales depending on the institution (Table 3).

Comparison of the variables with the scores obtained for these scales, using the multivariate logistical regression model, yielded the following results.

- For each unit increase on the GAF scale measurement, there was a 0.97-fold reduction in the risk suffering isolation (*P* = 0.054) and forced medication (*P* = 0.003) and a 0.96-fold drop in the risk of suffering restraint (*P* < 0.0001).
- For each unit increase on the MOAS, there was a 1.2-fold increase in the risk of suffering restraint (*P* = 0.027), while the 1.16-fold increase in the risk of suffering forced medication (*P* = 0.051) approached statistical significance.
- For each unit increase on the BPRS, the risk of suffering restraint was reduced 0.95-fold (*P* = 0.011).

Turning next to the patients' own perceptions, the most striking findings with regard to the subjective perception of coercive treatment were as follows.

Seventy-six patients (36.36%) stated that they had never previously been subjected to any coercive measure, while the remainder (133 = 63.64%) reported earlier experiences (67 once, 26 two or three times, and 40 >three times). Of the 171 patients responding to the question regarding the degree of pressure to which they were subjected (on a Cantril scale from 0—none—to 10—maximum—), the mean score was 5.51 (SD = 3.59).

*CAT Scale*. Analysis of patient perceptions of their treatment (scoring each of the items on a scale from 0 to 10) yielded the following results (Table 4).

With regard to the health professionals' perception of the coercive measures employed, the following observations can be made.

The perception of coercion through particular measures (on a scale from 0—none—to 10—maximum—) yielded a mean score of 6.3 (SD = 3.32).

The grounds given for implementation of the coercive measure were, in decreasing order of frequency, prevention of self-directed aggression (60.29%), prevention of aggression towards others (59.33%), need for treatment (22.01%), serious risk to their health (11.96%), prevention of material damage (11%), inability to care for themselves (6.22%), and patients' own request (1.91%).

The reasons for administering the measure were explained to most patients (90.3%), and in the vast majority of cases (91.9%) the episode was recorded in the case history, although a report was sent to the judicial authorities in only 126 cases (60.3%).

Finally, analysis of the degree of agreement between the patients' and the health professionals' responses revealed a moderate level of agreement on measures involving isolation (84.5% agreement, Kappa 0.6453, *P* < 0.0001) and physical restraint (82.44% agreement, Kappa 0.6402, *P* < 0.0001) and less, although also still significant, agreement with regard to forced medication (70.44% agreement, Kappa = 0.3849, *P* < 0.0001).

## 4. Discussion

Very little research has addressed the use of coercive therapeutic measures in the penitentiary context, as the EUPRIS report [11] points out. For that reason, this study sought to explore the patterns associated with coercive measures in psychiatric patients, with a view to minimising their use as much as possible. The methodology and variables employed here were similar to those of the EUNOMIA study [9], but adapted to the penitentiary context. One major resulting difference was that the present study ignored issues relating to involuntary admission, since all the patients were inmates, either of secure psychiatric hospitals or of regular prisons, and therefore admitted against their will.

Given the study design, it is not possible to comment on the prevalence of coercive measures, or even on their frequency of use, since no attempt was made to collect data on all the episodes taking place in each institution during the study period. Each researcher recorded cases of coercive measures employed with their own patients or with others of whom they happened fortuitously to be aware. Nonetheless, it would seem that the use of coercive measures on psychiatric patients in the various institutions declined over the study period. There is no statistical evidence in support of this assertion, although it does fall within reasonable expectations. Three factors may have contributed to this decline. The first was a visit paid to Spanish Penal Institutions by a delegation from the European Committee for the Prevention of Torture and Inhuman or Degrading Treatment or Punishment (CPT) between 19 September and 1 October 2007; in the course of the visit, the delegation showed interest in—among other issues—the use of restraint in the penitentiary context [16]. The second factor was the publication of rule 18/2007 by the General Directorate for Penal Institutions on “mechanical restraint,” which appeared just as the present study was starting, having been previously authorised; the rule has since then served as a form of protocol which, over time, has helped to reduce episodes of mechanical restraint on medical grounds. It fills what had previously been a gap in the regulations, as another author has pointed out [17]. The third factor is that, in itself, the systematic collection of data on cases involving the use of some kinds of therapeutic coercive measure has prompted greater attention to the criteria for implementing such measures and has led to a decline in their frequency; that is, it is an example of action research. Although not comparable, a study by D'Orio et al. [18], following the implementation of a plan based on the early identification and correct management of problem behaviour (mainly using a phased reduction of verbal input and continuous monitoring by video cameras) also achieved a 39% reduction in isolation and restraint episodes.

The most widely used coercive measure was isolation (41.35%), although in most cases (87%) it was accompanied by other measures (most commonly transfer or change of cell and forced medication). These percentages differ from those reported in the EUNOMIA study [9], where forced medication was the most frequent measure (56%), followed by restraint (36%) and isolation (8%). In evaluating these differences, it must be borne in mind that when patients are immobilised in a penitentiary setting they are nearly always isolated, and additionally they are usually held in rooms with video surveillance to enable constant visual monitoring. This viewpoint fits with the preference on the part of medical staff for isolation, as reported by Bergk and Steiner [19], because it was deemed less restrictive of human rights.

The present study found clear differences between types of institution in terms of the likelihood of suffering certain coercive measures; specifically, patients in secure psychiatric hospitals were more likely to suffer isolation and restraint than those in regular prisons. This would appear reasonable and probably reflects differences in patient pathological profiles depending on the institution (in our sample there were a greater proportion of psychotic and a lower proportion of substance-related disorders in the secure hospitals).

Hardly any statistically significant correlations were observed between personal, sociodemographic, or health variables and the type of coercive measure used; exceptions were whether the patient had lived with a partner prior to imprisonment (reducing the likelihood of being subjected to forced medication) and was in communication with family members (reducing the likelihood of being subjected to isolation measures).

These striking findings have not hitherto been reported in the literature, although it is widely recognised that marital status and social/family support point to a good prognosis for schizophrenia [20, 21]. There appears to be a straightforward explanation; in both cases, family and social support functions as a protective factor. Living with a partner requires a greater degree of social adaptation, because if psychosocial functioning is impaired it has an effect on the individual's roles as worker, student, partner, and family member and on personal care [22].

These findings clearly highlight the importance of encouraging family support for the wellbeing of these patients clear.

Previous custodial sentences and episodes of (nonpenal) psychiatric hospitalisation were both associated with a greater likelihood of being subjected to forced medication. However, previous imprisonment lowered the risk of being subjected to isolation and physical restraint. The first of these findings, at least with regard to previous experience of psychiatric hospitalisation outside the prison system, seems reasonable and matches published data suggesting a significant association with the administration of forced medication; diagnoses of schizophrenia, bipolar disorder, psychosis, and involuntary hospitalisation appear among the most common antecedents [23]. The role of previous imprisonment as a factor in avoiding isolation and restraint is more difficult to explain, unless it reflects a learning process on the part of inmates, whereby they become familiar with the way the institutions work and adapt to avoid such measures.

The psychometric scales used in the study were required to evaluate the health status of the sample population. Specifically, the global assessment of functioning (GAF) scale has been included in the DSM as a differentiating axis (axis V) and is the most widely differentiated measure for evaluating psychosocial deterioration. The mean score for participating patients was 54.19, which in clinical terms equates to moderate symptoms and moderate difficulties in the social and work spheres. Reasonably enough, higher scores on the scale (superior functioning) correspond to a reduced likelihood of suffering isolation, restraint, and forced medication, with the present study showing statistical significance in all three cases.

On the modified overt aggression scale (MOAS), the patients' mean score was 3.19 and the subscale with by far the highest scores was verbal aggression (mean = 1.32). The subscale scores do not tally well with the main reason put forward by clinicians for using coercive measures, that is, to prevent self-harm (60.29%), since the mean score for the self-harm subscale was 0.69 (on a scale from 0 to 4). Not surprisingly, a clear association was found between MOAS scores and two of the coercive measures: the higher the score, the greater the likelihood of being subjected to restraint (*P* = 0.027), while the increased likelihood of being subjected to forced medication approached statistical significance (*P* = 0.051).

The mean score on the BPRS was 48.15, indicating a population with pronounced psychopathology; the values were only slightly lower than those of a sample of 565 individuals with schizophrenia or schizoaffective disorders with a mean score of 50.05 [24]. Findings using BPRS were less unequivocal, though an increase in the BPRS score slightly reduced the likelihood of being subjected to restraint. This is in contrast to the results observed in the EUNOMIA study [9], where a diagnosis of schizophrenia and high scores on the BPRS were significantly correlated with the use of coercive measures.

Additionally, we found different profiles of results for the psychometric scales depending on the institution. It is important to outline that patients from regular prisons (RP) had lower scores in the GAF scale while had higher scores in MOAS and BPRS than in patients from secure psychiatric hospitals (SPH). All these data may suggest worse clinical condition in psychiatric patients from RP, probably due to a lower psychiatric monitoring of the illness, done by less specialized professionals and/or a higher rate of drug consumption.

With regard to patient perceptions, most patients (63.64%) had already been subjected to coercive measures in the past (half of them more than once). Their average perception of coercion fell exactly halfway up the Cantril “ladder” scale (from 0 to 10), with a mean score of 5.51; their evaluation of their treatment (CAT scale) was very similar, with mean scores for each of the scale's seven items slightly above the halfway mark (the highest score, 6.11, being for cordial relations with staff and the lowest, 5.65, for their view of the suitability of the medication received).

There is a certain degree of agreement between the patients' perception of coercion and that of the medical staff, the latter scoring even higher on this scale (mean 6.3). Lidz et al. [25] interviewed patients who identified health professionals as the most coercive group at the time of admission to hospital, stating that both verbal and physical coercion were used to pressurize patients into accepting hospitalisation. This is certainly the group exerting most pressure on patients, at least in terms of the therapeutic measures deemed necessary. Even so, considering that patients not only suffer coercive measures but also are inmates in a penal institution against their will, their opinion of health professionals and their evaluation of the treatment received were not excessively negative (the mean score for the relevant items was always higher than the midpoint of the scale).

There was also a fair degree of agreement between patients' and carers' perceptions with regard to the isolation and restraint measures employed, though they differed more with regard to forced medication (70.44% agreement). One explanation for this might be that the concepts of isolation and restraint are much more straightforward, with little room for disagreement, while the perception of forced medication is more likely to vary, being overemphasised by the patient and underemphasised by the clinician. Data obtained by the present authors in an as-yet-unpublished qualitative study of patients' and carer's views of such coercive measures also suggest that the measure prompting the greatest disagreement was the use of forced medication. Generally speaking, patients neither understood nor accepted it, whilst carers considered it wholly necessary. Patients expressed the view that, whilst accepting the use of coercive measures in specific circumstances, they preferred physical restraint to forced medication (especially in injectable form) due to the side effects produced by the latter. These opinions are somewhat more negative than those reported by Jarrett et al. [23] in their review of forced medication, which concluded that, with hindsight, many patients approved the use of forced medication, despite often negative experiences during the coercive episode.

A study carried out by Castille et al. [7], using the Mac Arthur perceived coercion scale, found no significant differences in the perception of coercion between patients following a compulsory outpatient treatment regime (assisted outpatient treatment, *n* = 76) and those following a non-compulsory outpatient regime (outpatient treatment, *n* = 108). The authors noted that the perception of coercion was associated with illness severity markers (e.g., low insight and number of previous hospitalisations) and feelings of being stigmatised.

According to Jarrett et al. [23], the most common reasons for forced medication, include the patients' refusal to take medication, were verbal aggression, agitation, and incidents-threats against medical staff, although Greenberg et al. [26] state that 43% of the 30 patients studied who received forced medication did so as a result of their plan of treatment, rather than because of any incident.

The frequency with which episodes were recorded in case histories (91.9%) leaves scope for improvement; ideally, such episodes should always be recorded. Though only 60.3% of episodes were notified by health services to the judicial authorities—which would appear to indicate considerable underreporting—this figure may be skewed, since in practice 37.8% of episodes were reported by the clinician in charge to the director of the institution, whose responsibility was to then notify the Supervisory Penitentiary Judge. Even so, it is probable that some cases of isolation and, especially, forced medication are not notified.

## 5. Conclusion

This is probably the most serious attempt made to evaluate factors related to coercive measures in psychiatric patients within penitentiary centers and secure psychiatric hospitals. Patients subjected to a coercive measure exhibited a pronounced psychopathology, as indicated by the percentage of psychotic disorders, scores on the BPRS and GAF scales, and the fact that most of them had been subjected to such measures on previous occasions. This finding prompts questions about the system itself and highlights the need either for more forensic psychiatric centres to house these inmates or for the appointment of psychiatrists to the specialist units planned for regular prisons. Few differences were found with respect to the type of coercive measures employed when comparing regular prisons with secure psychiatric hospitals, although there is a somewhat greater likelihood of being subjected to isolation and restraint in the latter, probably depending to a large extent on the patients' psychopathological profile and circumstances. The mere fact of external assessment of compliance with human rights slows down the incidence of coercive measures.

The limitations of the present study stem from the sample size, which was not large, from the design and scope, which impeded the systematic and random collection of all the coercive measures experienced by all patients in each institution, and lastly from the vulnerability and the difficulty in obtaining consent from the patients involved. Because of their dual status as patients with psychiatric disorders and as prisoners may have rendered subjects more vulnerable [27], the range of coercive factors to which they were exposed may potentially have affected the subjects' ability to provide informed consent. In any event, the present study was merely observational, showing the utmost respect for patients' rights and aimed at improving and minimising the coercive measures applied, in accordance with Recommendation 10 of the Council of Europe's Committee of Ministers to member states concerning the protection of the human rights and dignity of persons with mental disorder, dated 22 September 2004.

## Figures and Tables

**Table 1:** Patients' main characteristics.

	*n*	%
Gender		
(i) Men	181	86.6
(ii) Women	28	13.4
Age (years)		
Mean ± SD	34.5 ± 8.41	
Nationality		
(i) Spanish	188	90
(ii) Non-Spanish	21	10
Provenance		
(i) Penal institution	101	48.3
(ii) Secure psychiatric hospital	108	51.7
Main diagnoses		
(i) Dual pathology	80	38.3
(ii) Psychotic disorders	59	28.2
(iii) Substance-related disorders	16	7.6
(iv) Personality disorders	25	12
(v) Other psychiatric disorders	29	13.9
Marital status		
(i) Married or with a partner	25	12
(ii) No partner	184	88
Previous custodial sentences served*		
(i) Yes	162	79
(ii) No	43	21
Previously admitted to a secure psychiatric hospital*		
(i) Yes	130	66
(ii) No	67	34
Previously admitted to a psychiatric hospital*		
(i) Yes	131	65.5
(ii) No	69	34.5

*The actual sample was smaller due to the nonresponse of patients' or medical staff.

**Table 2:** Multivariate statistical analysis of variables related to coercive measures.

Variable	Type of coercive measure	Risk	According to patient report (odds ratio; *P*)	According to health professional report (odds ratio; *P*)
Type of institution (regular prison)	Isolation	Reduced	OR = 0.03; *P* = 0.026	OR = 0.26; *P* = 0.022
	Restraint	Reduced	OR = 0.06; *P* < 0.0001	
Previous custodial sentences served	Isolation	Reduced	OR = 0.12; *P* = 0.019	
	Restraint	Reduced	OR = 0.17; *P* = 0.001	OR = 0.15; *P* = 0.001
	Forced medication	Increased		OR = 8.34; *P* = 0.016
Previously admitted to psychiatric hospital (not secure)	Forced medication	Increased		OR = 4.3; *P* = 0.043
Living with a partner	Forced medication	Reduced	OR = 0.3; *P* = 0.071	
In communication with his/her family	Isolation	Reduced		OR = 0.32; *P* = 0.035

**Table 3:** Results obtained for the psychometric scales used to evaluate patient mental status in secure psychiatric hospitals (SPH) and regular prisons (RP).

	SPH			RP			Statistical differences
	Mean	SD	Median	Mean	SD	Median	
GAF	61.96	19.15	61	45.73	22.98	50	Yes, *P* < 0.001
BPRS	40.55	11.22	39	57.96	14.09	56	Yes, *P* < 0.001
MOAS	2.60	2.69	2	4.12	3.50	4	Yes, *P* = 0.001

**Table 4:** Results of the patients' scores of CAT scale.

Variable (scores from 0—no, not at all—to 10—yes, and completely—)	Mean ± SD
Consider the treatment received appropriate	5.89 ± 3.22
Staff take an interest in treatment	5.93 ± 2.98
Relations with staff are cordial	6.11 ± 2.62
Consider medication received appropriate	5.65 ± 3.31
Consider other aspects of treatment received appropriate	5.66 ± 3.29
Feel well regarded	5.70 ± 2.97
Helped by treatment	5.69 ± 3.20
